# High-throughput time series expression profiling of *Plasmopara halstedii* infecting *Helianthus annuus* reveals conserved sequence motifs upstream of co-expressed genes

**DOI:** 10.1186/s12864-023-09214-7

**Published:** 2023-03-21

**Authors:** Sakshi Bharti, Sebastian Ploch, Marco Thines

**Affiliations:** 1 Senckenberg Biodiversity and Climate Research Centre (SBiK-F), Senckenberg Gesellschaft für Naturforschung, Senckenberganlage 25, 60325 Frankfurt Main, Germany; 2 Department of Biological Sciences, Institute of Ecology, Evolution and Diversity, Goethe University, Max-von-Laue-Str. 9, 60323 Frankfurt Main, Germany; 3 Integrative Fungal Research Custer (IPF), Georg-Voigt-Str. 14-16, 60325 Frankfurt Main, Germany; 4 LOEWE Centre for Translational Biodiversity Genomics, Georg-Voigt-Str. 14-16, 60325 Frankfurt am Main, Germany

**Keywords:** Computational biology, Downy mildew, Gene expression, Obligate biotrophs, Oomycetes, Promotors, Transcriptional regulation

## Abstract

Downy mildew disease of sunflower, caused by the obligate biotrophic oomycete *Plasmopara halstedii,* can have significant economic impact on sunflower cultivation. Using high-throughput whole transcriptome sequencing, four developmental phases in 16 time-points of *Pl. halstedii* infecting *Helianthus annuus* were investigated. With the aim of identifying potential functional and regulatory motifs upstream of co-expressed genes, time-series derived gene expression profiles were clustered based on their time-course similarity, and their upstream regulatory gene sequences were analyzed here. Several conserved motifs were found upstream of co-expressed genes, which might be involved in binding specific transcription factors. Such motifs were also found associated with virulence related genes, and could be studied on a genetically tractable model to clarify, if these are involved in regulating different stages of pathogenesis.

**Supplementary Information:**

The online version contains supplementary material available at 10.1186/s12864-023-09214-7.

## Background

Plant pathogens have a diverse range of intracellular and extracellular effectors for host colonization and nutrient acquisition [1]. Pathogens secrete effector proteins to evade the host immune system and to obtain nutrients from host tissue. Biotrophic pathogens acquire nutrients from the living host, while necrotrophic pathogens kill host cells in order to access nutrients [1]. Oomycetes are fungus-like, eukaryotic microbes closely related to heterokont algae, i.e. the *Phaeophyta*, *Xanthophyta*, *Chrysophyta*, and *Bacillariophyta* [2–4]. Third to viruses and *Fungi*, oomycetes are the most important group responsible for plant diseases [5]. The majority of notorious plant pathogenic oomycetes belong to the genus *Phytophthora* (over 200 species) and to the downy mildews (over 700 species) [4, 6].

The highest species diversity of oomycetes is in the hemi-biotrophic and biotrophic parasites of flowering plants, including several important crops [7]. Obligate biotrophs, such as *Plasmopara halstedii* maintain a long-lasting interaction with their hosts [1, 8]. The genome assemblies of *Pl. halstedii* and *H. annuus* available in public repositories are 75.3 Mb and 3.6 Gb in size, and consist of 15,707 and 83,177 gene transcripts (both coding and non-coding genes), respectively [9, 10]. The sunflower downy mildew pathogen, *Pl. halstedii*, proliferates in *Helianthus annuus* as vegetative intercellular hyphae, which colonize the host leading to symptoms, i.e. systemic infection leads to dwarfing and chlorosis of leaves, and infected flowers produce abnormal infertile seeds, leading to oil yield losses [8, 11].

Transcriptional regulation impacts cellular processes in regulating cellular mRNA levels during developmental stages [12, 13]. The most well-understood control of gene expression in eukaryotes is the regulation of mRNA transcription with RNA polymerase II by regulatory proteins called transcription factors [14]. Sequence-specific transcription factor (TF) proteins bind with their transcription factor binding sites (TFBS), affecting the binding affinity of RNA polymerase upstream of the coding sequence [15].

In eukaryotes, each gene has several functional elements, e.g. core promoter, proximal and distal promoter sequences, as well as regulatory motifs in the 5’UTR close to the transcriptional start, each with specific sequence motifs recognized by sequence-specific TF protein families [16]. However, this knowledge is mainly derived from animals, yeast, and flowering plants and little is known about the genetic basis of transcriptional regulation in oomycetes. Most of the work has been focused on the core promoter sequence sites at which the transcription initiation complex is assembled [17, 18]. The core promoter consists of two important elements, a TATA box or another motif of similar function located 25–30 nucleotide upstream of the TSS and the transcription start site (TSS) or INR or “the +1 position” that is often represented by YYANWYY in humans and TCAGTY in *Drosophila melanogaster*, even though it should be noted that in humans, only a minority of promoters contain a TATA-box or other simple motifs, necessitating detailed bioinformatics analyses for determining their structure and function [19]. In oomycetes, T-rich conserved motifs with a probably TATA-box like function are located 50–100 nucleotides upstream of start codon [20]. Very little is known about the genetic basis of transcriptional regulation in oomycetes. Whole-genome analysis revealed the presence of extended FPR (MWTTTNC) motif surrounding the INR (YCAYTYY) or TSS, AT-rich represent functional non-canonical TATA-like elements with weak positional preference and DPEP motif (SAASMMS) located 25 nucleotides downstream of the TSS [21–23]. In an earlier study the genome of the sunflower pathogen *Pl. halstedii* has been sequenced [10] and a positional bias of INR + FPR supra-motifs and CCAAT within oomycete promoters has been found [10, 24]. Another study identified the conserved core effectors in 17 Pl. halstedii pathotypes potentially targeting the host organelles and thereafter validated from inducing hypersensitive response in host sunflower broad spectrum resistant lines [25]. Studies in *Plasmodium falciparum* have shown that strong motif groups can be identified in the upstream regions (< 2000 nt of translational start site) of 13 sets of genes with a similar expression pattern suggesting functional coordination [26].

Illumina-based RNA sequencing technologies provide quantitative gene expression data and, thus, a basis for studying transcriptional regulation by grouping genes with similar expression patterns [27, 28]. Chromatin immunoprecipitation sequencing (ChIP-seq) experiments are a common method for identifying binding sites and their specific transcription factors, but ChIP-seq has limited potential for the identification of novel transcription factors as they rely on TFs that are not sequence-specific [29, 30]. Thus, RNA-seq might be a solution to identify novel regulatory sequences by associating TFBS with their downstream genes. Clustering algorithms can be used to identify gene expression changes over time, as similar expression patterns are likely to be the result of regulation by the same transcription factor family [31]. An example using this approach is a recent study on *Listeria monocytogenes* [32]*.* The study based on RNA-seq data from *L. monocytogenes* found a previously unreported partially palindromic motif M58.7 in the promoter regions of ribosomal protein encoding genes that regulate the growth of bacteria [22]. De novo DNA motif discovery analysis in the late blight pathogen *Ph. infestans* and two other *Phytophthora* species (*Ph. ramorum* and *Ph. sojae*) identified 19 conserved DNA motifs that correlated with gene expression levels during infection [33]. In another study, gene-expression changes in both *H. arabidopsidis* and its host *Arabidopsis thaliana* during infection were investigated in compatible and incompatible interactions, and identified some promoter elements associated with *H. arabidopsidis* genes [24]. However, the studies only involved a few time points and were not focused on a genome-wide analysis of transcriptional changes.

In this study, the transcriptional changes during the infection of sunflower with *Pl. halstedii* were investigated by analyzing shifts in mRNA expression profiles during developmental transitions (zoospore release, establishment of infection, colonization, and induction of sporulation). The aim of the study was to investigate the hypothesis that the upstream regulatory sequences of co-expressed genes might have discernable overrepresented motifs in core promoter regions close to the coding sequences. For this, we chose to analyse the motif-rich stretch 100 bp upstream of genes that had been identified as containing the core promoter in previous studies, even though we are aware that many promoters will not be fully covered by this approach and that longer upstream sequences would likely have revealed additional motifs.

### Experimental design, materials and methods

The study complies with relevant institutional, national, and international guidelines and legislation for plant ethics.

#### RNA isolation, library preparation and transcriptome sequencing

Approximately 250 sunflower seeds (*Helianthus annuus*, cultivar Giganteus) were moistened for 36 h, peeled, and potted into gardening soil (Gärtnerpflanzerde, Hornbach, Germany). The plants were grown at 16 °C until the cotyledons were fully developed, carefully taken out of the soil maintaining the roots, and washed with distilled water twice to remove residual attached soil. The plants were kept overnight in a moist chamber. In parallel, sporulation of *Plasmopara halstedii* was induced in six systemically infected plants by placing them in a moist chamber into darkness overnight at 12 °C.

#### Infection of seedlings and zoospore release phase (time points 5 min, 15 min)

The sporulating plants were removed from the moist chamber, left to dry for about 45 min before the sporangium solution was prepared by cutting the leaves off and thoroughly rinsing them in approximately 2 L of autoclaved, distilled water. Triplicate samples were taken after 5 min and 15 min by transferring 25 ml of the inoculation solution in a 50 mL tube and immediately adding 25 mL of 2-propanol (100%). The tube was centrifuged for 1 min at 6000 *g*. The supernatant was removed until 5 mL remained, the residual sample was resuspended and transferred into a new 15 mL tube to which an equal amount of RNAlater (ThermoFisher Scientific, Waltham, USA) was added. The tube was centrifuged as described above and the sporangia that accumulated in the interphase between RNAlater and 2-propanol were carefully pipetted off and transferred to a new 1.8 mL cryotube, which was filled up with RNAlater. All tubes were placed into the − 80 °C freezer immediately after sample collection was completed. After 60 min the rinsed seedlings were added to the sporangia solution and incubated in darkness at 16 °C.

#### Establishment of infection (time points 4 h, 8 h, 12 h, 24 h, 48 h, 72 h)

The time points covering the establishment phase were sampled by scraping off the surface of the cotyledons to enrich material from the pathogen. To ensure RNA integrity, the cotyledons were dunked into RNAlater™ solution and the epidermal layer of the cotyledons were immediately scraped off from the upper- and lower-leaf surface with a scalpel blade. The material was then transferred to a 1.8 mL cryotube containing 1 mL of RNAlater. In addition, the scalpel blade moistened with RNAlater before, after, and in-between the scraping of every cotyledon. In total four plants (with two cotyledons each) were scraped per sample, and three independent samples were taken.

After 8 h, the remaining seedlings were taken out from the inoculation solution and placed in a closed moistened plastic box. After 48 h all seedlings, except for those to be sampled at time-point 72 h, were potted into fresh gardening soil in a climate chamber of the mesocosm hall (Senckenberg Biodiversity and Climate Research Centre, Frankfurt, Germany), which was set to a daily rhythm with 14 h light at 16 °C and 70% relative humidity and 10 h darkness at 12 °C and the same humidity. The day/night cycle was inverted to the natural day/cycle so the plants were always sampled at the end of their day.

#### Colonization phase (time points 120 h, 222 h, 288 h)

Soil attached to the seedlings was rinsed off using autoclaved, deionized water. For each sample three seedlings were sampled by cutting a square of approximately 10 mm from the central part of each cotyledon with a sterile scalpel blade and transferring this to a 1.8 mL cryotube filled with RNAlater. The sample was immediately disrupted using a blunt pistil to ensure RNA preservation of the whole sample.

#### Induction of sporulation (time points 290 h, 292 h, 294 h, 296 h)

All residual plants not sampled at time-point 288 h, were cut, rinsed with autoclaved, distilled water and placed into a moist chamber in darkness to induce sporulation. Time-points 290 h and 292 h (induction of sporulation) were collected as described for the colonization phase but without rinsing the cotyledons, and sampling four plants instead of three per replicate. Time-points 294 h and 296 h (sporulation phase) were sampled similarly to the establishment phase by scraping off the epidermis with a sterile scalpel blade but only in areas with visible sporulation. Since visible sporulation was not only present on the cotyledons but also on the primary leaves, an additional three samples per replicate were taken at time point 296 h by scraping off the lower leaf surface of primary leaves that showed sporulation as described above.

#### RNA extraction

The RNA was extracted from the RNAlater-preserved material using a NucleoSpin RNA Plant Mini Kit (Macherey-Nagel, Düren, Germany) according to the manufacturer’s instructions. RNAlater solution was removed prior extraction by filtration (samples with low amounts of tissue) or centrifugation (samples with high amounts of tissue). For filtration, the samples were filtered through the sterile filter of a filter-tip (Biozym, Hessisch Oldendorf, Germany), the filter removed aseptically, and the upper half millimetre of the filter with the desired material cut and homogenized using metal beads together with lysis buffer as described in the manual to the kit. Library preparation and transcriptome sequencing (illumina 100 bp paired end) was conducted by Eurofins Genomics (Luxemburg).

#### Transcriptome analyses

**Fig. 1:**
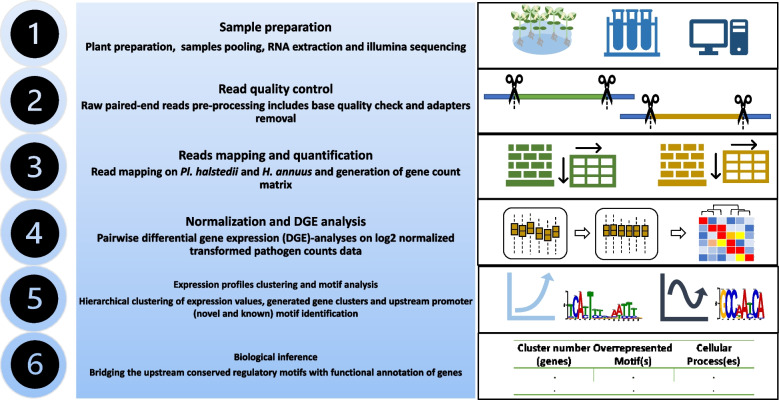
Flowchart of the experimental design for RNA-seq and data analysis

A flow-chart showing the methodology followed to prepare RNA sequencing data and to conduct downstream data analysis is given in Fig. 1.

#### Read preprocessing, quantification, normalization

The raw paired-end RNA-seq reads were processed using Trimmomatic [34]. Standard sequencing primer and adapter sequences (parameters ILLUMINACLIP: TruSeq3-PE. fa:2:30:10:8:true) were trimmed and reads with an average Phred score less than 33 (−phred 33) and reads shorter than 20 nt (SLIDINGWINDOW:5:20 LEADING:3 TRAILING:3 MINLEN:30)were removed. The STAR mapper [35] was used for mapping the processed reads to both host (HanXRQr1.0, Ensembl Plants release 49 [9];) and pathogen (Plasmopara_halstedii_gca_900000015.1, Ensembl Protists release 49 [10];) gene transcripts including coding and non-coding genes. Initially, the reference index for the genomes was generated using the STAR mapper and later sequenced reads were mapped using the reference index. To perform quantification analyses, filtered reads were analyzed by FeatureCounts [36] of the Subread package v2.0.1. FeatureCounts accumulates gene count information by mapping reads to the exonic regions of genes (−t exon) and the parameter for reads overlapping in multiple transcripts were selected. Reads that mapped uniquely to the pathogen genome were used for downstream analyses and only locations with 10-fold or higher coverage were retained. As part of expression normalization, within-sample and between-sample normalization was performed across all 16 time points and their three biological replicates, each.

#### Differential gene expression analysis

To account for over-dispersion or high variability of genes, negative binomial distribution for each gene and gene-wise dispersions were estimated. Expression levels and Z-scores were calculated across all 48 samples (16 time-points) by subtracting the observed value from the mean expression level of the time-point and dividing this by the standard deviation following pairwise comparisons between time-points [37]. The DESeq2 likelihood ratio test was conducted using False Discovery Rate corrected *p*-values or adjusted p-values (p-adj) ≤ 0.01 to identify highly significant differentially expressed genes between the time-points [38].

#### Expression profile pattern-based gene clustering

For gene clustering and visualization, after transformation hierarchical clustering was used to classify sets of genes with similar expression pattern on the basis of pairwise Kendall rank correlations (at least 15 genes per cluster, correlation > 0.7, testing the hypothesis of correlation for a confidence interval of 95%), and outliers from the cluster distribution were removed. For every clustered gene set, expression values are visualized in terms of Z-scores using DEGreport across the 16 time-points [39].

#### 5’UTR dataset and background model

With the aim to search for over-represented regulatory motifs in the upstream regions of each cluster of genes, upstream regions (100 nt) were extracted using python scripts from the reference genome of *Pl. halstedii* [14] and a core promoter sequence dataset was generated. All extracted sequences were oriented in 5-prime to 3-prime direction. Using the fasta-get-markov Perl script in the MEME (Multiple Expression Motifs [EM] for Motif Elicitation) suite 5.3.0, a 3rd order Markov background model file from all 5’UTR regions (core promotor regions, e.g. the stretch up to 100 bp from the start codon) FASTA sequences was generated [40].

#### Motif discovery

With the purpose of motif identification in core promoter regions located within 100 bp upstream of genes, MEME was used for clusters with less than 50 genes and STREME for clusters with more than 50 genes [41]. Motif lengths of 4 to 18 nt (e-value ≤0.5) were searched for, using the zero or one occurrence per sequence model in the sequences of each cluster. The binomial test based on differential enrichment was set to search for motifs with a *p*-value ≤0.05, with up to 20 iterations on the reference genome of *Pl. halstedii*. To each set of gene clusters and reference upstream regions, an empirical 3rd order background Markov model was applied, based on all upstream regions of the *Pl. halstedii* genome and accounting for single-, di-, tri- and tetra-nucleotide distributions for each set. The discovered motifs in the clustered genes were searched against the reference genome motifs database of *Pl. halstedii* generated by STREME. AME (Analysis of motif enrichment) of the MEME suite was used to detect whether the identified potential transcription binding site motifs were enriched (e-value ≤0.5) in set(s) of the upstream sequences, as defined by belonging to the same expression cluster, compared to the reference genome motif sequences [42]. To scan the sequences for individual matches to each of the enriched motif(s), FIMO (Find Individual Motif Occurrences) was used [43].

#### Inference of biological roles associated with enriched motifs

From the published genome of *Pl. halstedii*, genes annotated as pathogenicity related and those involved in core biological pathways were obtained from the supplementary data of Sharma et al. [10]. Furthermore, classifications based on the published functional annotations were done using shell scripts. Genes highly expressed, as inferred from the RNA-seq data, and annotated as encoding for transcription factor (TF)-proteins were compared to those in the list of known predicted transcription factors in oomycetes as represented in FungiDB [44].

## Results

### RNA-seq data pre-processing, mapping and identification of Pl. halstedii encoding transcripts

To infer differential expression during different infection stages of the obligate biotroph *Plasmopara halstedii* interacting with *Helianthus annuus*, a time-series analysis was done. This included zoospore formation (harvested zoosporangia; 5 min and 15 min after incubation in water), establishment of the infection on the cotyledons surface (IF; 4 h, 8 h, 12 h, 24 h, 48 h, 72 h), colonization phase (CO; 120 h, 221 h, 288 h) and sporulation phase (SP; 290 h, 292 h, 294 h, 296 h, sporulating primary leaves at 296 h) (Fig. 1). Forty-eight cDNA samples (three biological replicates per time-point) were done from the different stages of infection. The sequencing of the resulting 48 Illumina paired-end libraries resulted in 28.3 million to 34.6 million reads for each time-point. After filtering, 94.3 to 96.7% reads were used for downstream analyses (Table 1). These were mapped to the 15,707 predicted genes of *Pl. halstedii* and the 61,131 predicted genes of *H. annuus* using the splice-aware mapper STAR, and reads mapped to the host were excluded from the further analyses of this study (Additional files 1 and 2). The reads uniquely mapping to *Pl. halstedii* ranged from 0.19 million to 30.3 million. Of the 15,707 unigenes of *Pl. halstedii* 12,801 showed expression during the stages sampled (Additional file 3).

**Table 1:** Summary of read processing results for the various time-points in the four lifecycle phases with their respective time points

Pathogen growth phases	Time points	Range of passing reads (%)	Uniquely mapped reads pathogen (%)	Uniquely mapped reads host(%)
Zoospore release phase	T5min, T15min	94.2–95.3	90.2–95.1	0.02–0.49
Establishment of infection	T4h, T8h, T12h, T24h, T48h, T72h	95.0–96.2	0.7–3.85	83.9–88.9
Colonization phase	T120h, T221h,T288h	96.6–96.7	6.2–42.7	50.5–87.5
Induction of sporulation	T290h,T292h, T294h, T296h,T296h_prim	95.8–96.5	28–94.9	0.5–64.4

### Data variability, normalization and statistical analysis of differential gene expression

**Fig. 2:**
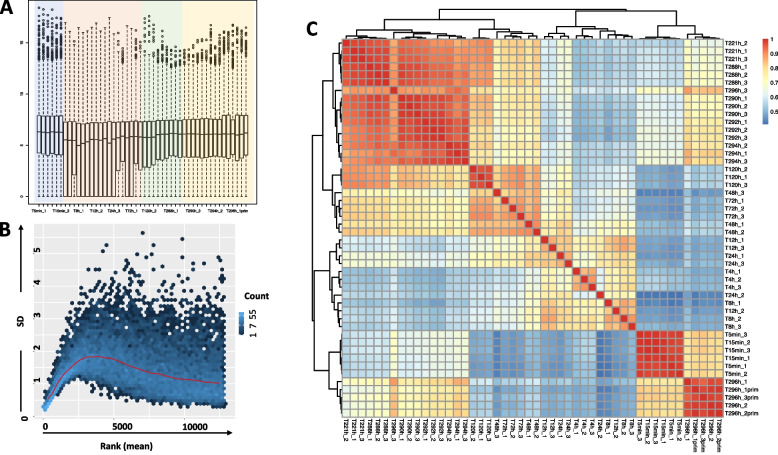
Overview on the expression data. **A** Normalization boxplot representing sequencing depth and rescaled log2 counts per million for 16 time points (16*3 biological replicates). **B** Gene-wise variance estimation, mean vs. standard deviation plot for normalized row-wise gene counts. The horizontal red line is the median estimator. **C** Variance-stabilized transformation of normalized raw counts and Pearson correlated clustering of expression patterns for the 100 most-expressed genes

Some genes had a higher variance than others, consequently their expression values varied more from one time-point to other. Differently expressed genes have been identified with the assumption of equal dispersion across all the time-points [45]. With mean and variance linked by local regression, the negative binomial model fits best to the pathogen count data as their mean is less than their variance. To account for variance bias between and within samples, scaling according to library size was used as a first normalization step (Fig. 2, Additional file 3).

After additional removal of gene data with zero or low expression change (log fold change < 0.58, or fold change 1.5) a likelihood ratio test with 15 pairwise comparisons of 16 time points 11,689 genes were found to be significantly differentially expressed (adjusted *p*-value < 0.01), which is about 90% of the overall genes expressed.

### Gene expression clustering

**Fig. 3:**
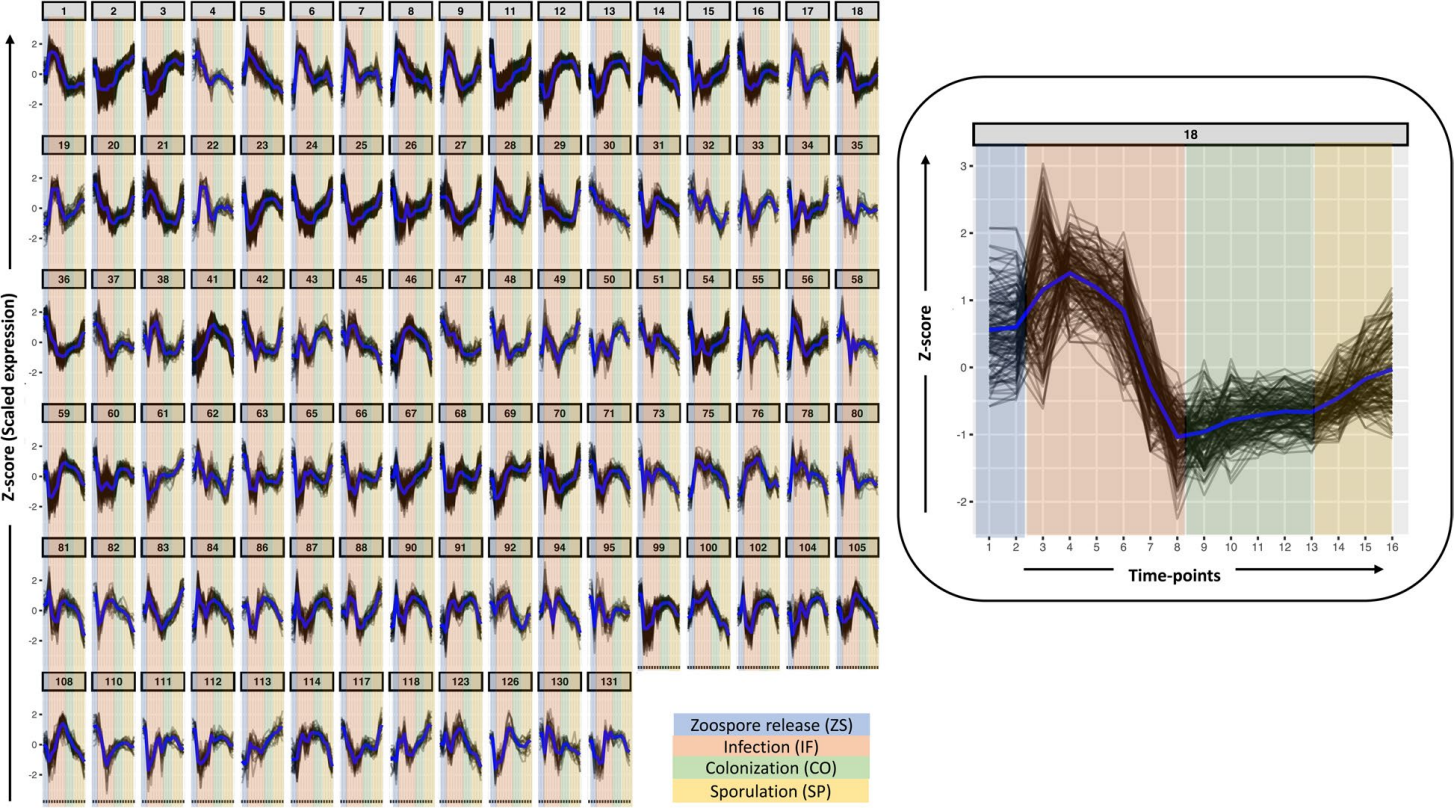
Clustered and scaled expression patterns for the top 500 differentially expressed (DE) genes. Each group represents a gene expression pattern shared among different DE genes

For identifying patterns among the 16 time points from the four infection phases, hierarchical clustering of the genes based on their transformed expression fold-change was done. Clusters of genes were generated by hierarchical clustering of a gene to gene expression distance matrix and separated using divisive coefficients of the clustering. Based on pairwise correlations and scaled expression, a total of 97 significant clusters with a minimum number of 15 genes per cluster, containing 8444 differentially expressed genes (Figs. 2c and 3, Additional file 3) were obtained.

### Core promoter motifs in the *Pl. halstedii* genome

For investigating putative core promoter reference motifs, the upstream 100 nt from the start codon ATG were used for all genes of *Pl. halstedii*. By this approach, 17 over-represented, statistically significant motifs (*p* < 0.05) were found. Except for streme-4 (TCATTYTYMAATTTK, 990 occurrences), streme-10 (GCCAAYCA; 209 occurrences), streme-13 (CAGCAACATC, 3114 occurrences), to the best of our knowledge, other motifs have not been reported previously from downy mildews. Among the novel motifs and streme-2 (CTTCCTCTTC, 1181 occurrences) had the highest occurrence as compared to the other 15 significant motifs (Table 2). Notably, there were no clusters identified representing the streme-2 (CTTCCTCTTC, 1181 occurrences), streme-14 (CACATCAA, 405 occurrences), and streme-16 (CGTCGACCCTGAYCC, 34 occurrences) motifs (Additional file 4), suggesting a more general function.

**Table 2:** Gene-rich clusters with their significant motifs (ID, sequence, sites/occurrences, logo), *p*-value < 0.05, identified from the *Pl. halstedii* reference genome using STREME. The remaining 9 STREME motifs are listed in Additional file 4. Motifs retrieved from MEME for each cluster are tabulated in Additional file 5

Motif (ID, sequence, sites)	Motif logo	P-value (<0.05)	Cluster IDs (No. Of clusters)	Highly abundant cluster(s) ID (No. of genes)
STREME-1, DRTGTGGWCCACAYH, 379		1.20E-07	11, 12, 13, 14, 23, 25, 31, 33, 41, 46, 58, 59, 63, 67, 92, 99,100,105, 114, 126, 131; (21)	11(62), 12(68)
STREME-3, GATTGGHTRAAAWDW, 484		9.20E-06	3, 5, 6, 11, 12, 13, 14, 18, 23, 24, 26, 33, 34, 41, 46, 51, 56, 59, 61, 65, 67, 69, 79, 86, 90, 91, 100,102,104, 105, 108, 111, 123, 126; (34)	11(55), 12(59), 41 (47)
STREME-4, TCATTYTYMAATTTK, 990		3.70E-05	1, 2, 3, 6, 7, 8, 11, 12, 13, 14, 18, 20, 21, 26, 27, 28, 29, 34, 35, 36, 41, 43, 59, 65, 66, 68, 70, 76, 78, 84, 86, 87, 90; (33)	11(76), 14(48)
STREME-6, ATGGCTACA, 49		1.90E-03	2, 11, 41, 66, 68; (5)	11(30), 2(16)
STREME-10, GCCAAYCA, 209		2.80E-02	8, 11, 12, 14,34, 41, 59, 63, 65, 68, 102, 105, 108; (9)	11(3), 14(13)
STREME-11, ARCCCCGYTRCA , 60		3.10E-02	17, 67, 131; (3)	67(3)
STREME-12, AACTTCAAC, 183		3.20E-02	3, 15, 23, 34, 54, 130; (6)	3(24), 54(11)
STREME-13, CAGCAACATC, 3114		3.20E-02	11; (1)	11(55)

### Core promoter region analysis of co-regulated and/or co-related genes within clusters and motif discovery

The 97 clusters of co-regulated genes contained 8432 genes and 16 to 1158 members. For an efficient motif discovery, the clusters were further divided into two categories based on their size. STREME, based on an advanced ab initio motif discovery algorithm, generates more accurate motifs for clusters with at least 50 genes [41]. Of the conserved motifs found using STREME on clustered gene data some matched with 17 known regulatory motifs. Based on de novo motif discovery in smaller clusters using MEME, 61 statistically significant motif(s) were found for 53 out of 97 clusters varying from 4 nt to 16 nt (Table 2; Additional file 5).

### Pathogenicity related genes

**Fig. 4:**
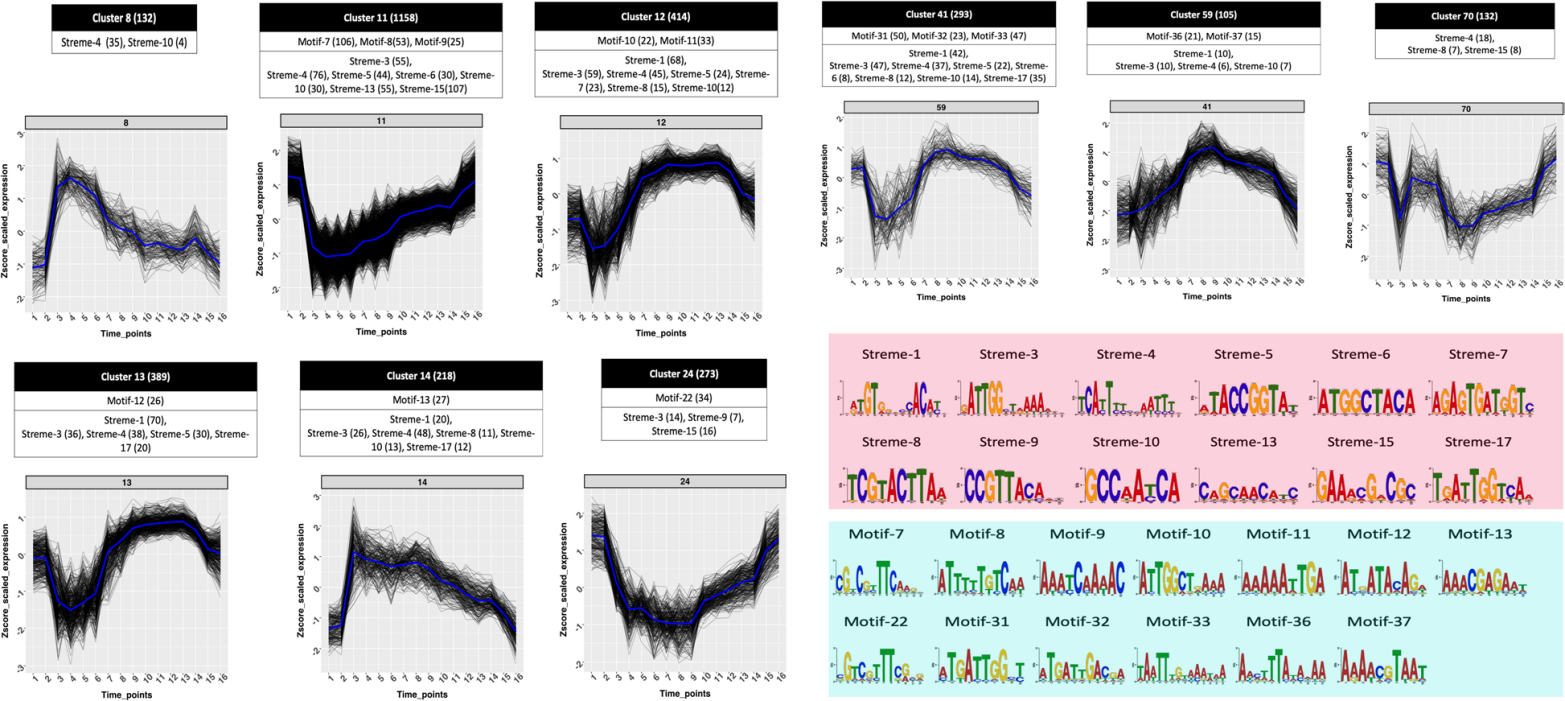
Putative regulatory motifs for clusters enriched in genes associated with pathogenicity, exocytosis and vesicle transport, ion channels and calcium binding proteins, plant cell wall degrading enzymes and transcription factors. The X-axis in the expression profile shows 16 time-points of *Pl. halstedii* lifecycle and on the Y-axis the gene expression scaled according to Z-score. The number shown above every graph gives the cluster number from the current study. The identified motifs are given below each of the expression clusters. Known motifs are shaded in pink, new motifs in turquoise

Some highly supported motifs were found for clusters which are highly enriched with genes related to pathogenicity, exocytosis and vesicle transport, ion channels and calcium binding proteins, plant cell wall degrading enzymes and transcription factors. These are shown in Fig. 4, along with their expression profiles.

In total, 381 putative secreted effector encoding genes were expressed. Out of these 175 were annotated as RxLR effector candidates. In addition to this, 27 CRNs (Crinkler gene family), 103 secreted proteases, 13 protease inhibitors, and two genes coding for ATP-binding cassette (ABC) transporters were found in clusters with a similar expression pattern. The majority of the secreted proteases identified were serine-, aspartyl-, cysteine-, ubiquitin- and metallo-proteases (Table 3; Additional file 6).

**Table 3:** Distribution of pathogenicity-related genes over the different expression clusters

	Pathogenicity related genes				
	RxLRs	CRN (Crinkler gene family)	Secreted proteases	Protease inhibitors	ATP-binding cassette (ABC) transporters
**Number of genes**	175	27	103	13	2
**Cluster ID (Number of genes)**	1(7), 2(7), **3(9**), 5(2), 6(1), 7(1), **8(12**), 9(2), **11(14), 12(10**), **13(9), 14 (15),** 15(1), 17(1), 18(4), 20(2), 21(4), 22(1), 23(2), 24(4), 25(2), 27(1), 28(3), 36(4), 38(2), 41(12), 46(3),47(1), 56(2), 59(2), 60(1), 62(2), 63(1), 65 (1), 66 (2), 68 (1), 75(1), 76(2), 81(4), 84(3), 87(1), 90(5), 91(1), 92(2), 99(1), 100(2), 102(1), 114(1), 130(3)	2(2), 5(1), 7(1), **11(6)**, 16(1), 19(1), 23(1), 24(1), 25(1), 26(1), 34(1), 54(1), 60(1), 61(1), 65(1), 67(1), 69(1), 71(1), 91(1), 102(1), 105(1)	2(5), 3(5), 7(1), 8(6), **11(9)**, **12(13**),16(1), 18(5), 20(1), 21(1), 23(3), 24(4), 25(2), 26(1), 27(1), 28(2), 33(1), 36(2), 42(1), 46(1), 49(1), 51(1), 54(1), 55(1), 56(1), 59(3), 61(1), 62(1), 65(1), 67(3), 68(3), 69(4), 81(1), 88(1), 99(3), 105(2), 111(1), 112(1), 113(1), 126(1)	2(1), 4(1), 6(1), **8(2**), 11(1), 14(1), **16(2)**, 29(1), 35(1), 41(1), 59(1)	**12(1), 59(1**)

### Exocytosis and vesicle transport

Vesicle exocytosis, the fusion of a pathogen derived vesicle with the cell plasma membrane, releases vesicular contents to alter host immune responses [46]. In this study 37 genes involved in exocytosis and protein export from the endoplasmic reticulum to the Golgi apparatus were found to be differentially expressed during different stages of infection, including SEC1, SEC3, SEC4, SEC13, SEC14, SEC16, SEC23, SEC24, SEC61, some SNARE proteins, and Rab GTPases (Table 4; Additional file 7).

**Table 4:** Distribution of genes for exocytosis and vesicle transport, ion and calcium binding proteins, PCWDEs and transcription factors over the various expression clusters found. Numbers in bold indicate expression clusters that also contain effector candidates

	Exocytosis and vesicle transport	Ion channels	Calcium-binding proteins	Plant cell wall degrading enzymes (PCWDE)	Transcription factors
**Number of genes**	37	53	33	27	135
**Cluster ID (Number of genes)**	2(1), 3(2), 7(1), 9(1), **11(4), 12(4**), 13(2), 14(1), 19(1), 20(1), 21(1), 23(1), 25(1), 28(2), 36(2), 38(1), 41(1), 54(1), 59(1), 66(1), **70(3)**, 83(1), 95(1), 102(1)	2(1), 3(3**), 11(7),** 12(2), 13(1), 14(1), 16(1), 20(1), 23(1), **24(5)**, 25(3), 26(3), 27(2), 29(1), 36(2), 41(3), 42(1), 45(1), 46(1), 50(1), 51(1), 54(1), 56(1), 58(1), 69(1), 70(2), 78(1), 86(1), 99(2), 111(1)	2(4), 3(1), 8(1), **11(4)**, 13(2), 15(1), 16(1), 18(3), 20(2), 23(1), **24(6)**, 25(1), 30(1), 47(1), 60(1), 69(1), 71(1), 99(1)	2(1), 3(1), 6(1), 8(2), 11(1), **12(3),** 13(1), 23(1), **24(2)**, 25(1), 29(1), **41(3)**, 42(1), 46(1), 67(1), 69(1), 83(1), 84(1), 88(1), 90(1), 126(1)	1(1), 2(4), **3(12)**, 4(1), 7(4), 8(1), 9(1), **11(21)**, 12(2), 13(5), 14(2), 15(3), 16(1), 18(2), 20(2), 21(3), 23(3), 24(8), 25(2), 26(1), 27(1), 29(1), 30(1), 33(1), 36(1), 37(1), 41(4), 43(2), 45(1), 46(1), 49(1), 50(1), 51(1), 54(6), 58(1), 60(2), 63(2), 65(1), 67(1), 69(4), 70(1), 75(1), 83(1), 86(1), 87(2), 90(1), 94(1), 100(1), 102(1), 105(2), 108(1), 111(1), 112(1), 113(2), 114(1), 117(1), 123(1)

### Ion channels and calcium-binding proteins

Ion channels are specialized proteins, through which Na^+^, K^+^, Ca^2+^ and Cl^−^ ions are transported through membranes. They are possibly important countering early host signaling to induce immune responses, including apoptosis or programmed cell death [47]. There are in total 53 genes encoding for calcium-activated potassium channels, chloride channels, and voltage-gated ion channels differentially expressed throughout different lifecycle stages of the pathogen (Table 4; Additional file 8). Others differentially expressed ion channels include inward rectifier potassium channels (IRK), polycystin, and a sodium potassium-transporting ATPase subunit.

Calcium binding proteins (CBP) also act as Ca^2+^ transporters across cell membranes, thereby controlling Ca^2+^ concentration in the cytosol and regulating cellular functions. In response to a Ca^2+^ increase in the cell, CBP such as calmodulin and calcineurin indirectly regulate phosphorylation/dephosphorylation of transcription factors [48]. Most of the differentially expressed calcium binding genes (33 genes) transcribed were classified as calcineurin, calmodulin, EF-H superfamily, CDKs, dynamin and protease kinase inhibitors. (Table 4; Additional file 8). If the differentiation of the mostly endogenously-acting proteins mentioned above are needed for pathogenicity and serve homoeostasis throughout different lifecycle stages needs to be clarified in future studies.

### Plant cell wall degrading enzymes (PCWDEs)

Pathogens invade the host plant through the degradation of plant cell walls composed of polysaccharide components such as pectin, cellulose and hemicellulose [49]. In the current study, 27 potential genes for secreted carbohydrate-active enzymes (CAZyme) were found differentially expressed, including members of carbohydrate esterase (CE), glycoside hydrolase (GH), glycosyl transferase (GT) and polysaccharide lyase (PL) families (Table 4; Additional file 9).

### Transcription factors

Transcription factors (TFs) or sequence-specific DNA binding factors are the proteins that bind to the upstream regulatory elements of genes and stimulate or inhibit transcription. In the present study, 135 transcription factors were found to be differentially expressed during different life-cycle stages, primarily from the myb factor and zinc finger families. In oomycetes, the myb DNA-binding domain is involved in growth and developmental processes [50], and 23 myb TFs were found to be differentially expressed in this study. In oomycetes, the zinc finger proteins form one of the largest families of transcriptional regulators of growth, development and pathogenesis [51, 52]. In this study, 74 members of zinc finger and 18 members of the bZIP families were found to be differentially expressed. Other families found were helix-turn-helix (HTH; 5), general transcription factors (10), homeobox (3), and heat shock related (2) TFs (Table 4; Additional file 10).

## Discussion

Despite of recent insights into core promotors of oomycetes [10, 22], clues for motifs involved in regulation are largely lacking, with a few exceptions [33, 53]. In this study a systems-level exploration of the of the differential transcription of genes expressed throughout the lifecycle of *Plasmopara halstedii* was done. Overall, 97 clusters of similarly regulated genes were observed, which might be useful to understand the functionality of the un-annotated genes from other annotated genes in the same expression cluster [54].

### Non-coding RNA

There is evidence for differential regulation of long non-coding RNAs (lncRNAs) in *Ph. sojae* [55]. Relating to Jashni et al. [56], the current *Pl. halstedii* transcriptome study provides additional support to the presence of putative ncRNAs expressed at lower levels (28.5%) than coding RNAs. For the non-coding gene ENSRNAG00050153322 (U6atac minor spliceosomal RNA), the motif streme-12 (AACTTCAAC; *p*-value 6.37E-05; cluster 34) in the upstream DNA sequence has been found. This ncRNA was co-expressed with a gene cluster enriched with secreted crinklers, e.g. gene CEG41152 (CRN-like protein; LFLAK- Position: LYLAK-40), suggesting a potential function associated with effector regulation [55].

### Prominent motifs and in oomycete core promotors

The candidate regulatory motifs streme-4 (TCATTYTYMAATTTK; 15 nt) and streme-10 (GCCAAYCA; 8 nt) bear similarities to a combined INR (YCAYTYY) + FPR (MWTTTNC) and the CCAAT box, respectively, which were detected in the oomycetes *Ph. infestans*, *H. arabidopsidis, Ph. sojae, Globisporangium ultimum,* and *Saprolegnia parasitica* [21, 57] and, thus, seem to be general motifs involved in gene expression and transcriptional start in oomycetes. In the current study, the combined INR-FPR motif was detected 990 times (enrichment p-value 0.000037) in 33 clusters of the whole set (Additional file 4), while streme-10 (putative CCAAT box) was detected 209 times (enrichment p-value 0.028) in 9 clusters, suggesting a low conservation of the motif. The putative regulatory motif streme-4 (TCATTYTYMAATTTK; 15 nt) and streme-13 (CAGCAACATC; 10 nt), to some extent, shows similarity with predicted transcription start site (TSP1, flanking upstream of TSP2 respectively) in core promoters of *PinifC1*, *PinifC2,* and *PinifC3* genes of *Ph. infestans,* as well as putative homologues from *Ph. sojae* and *Ph. ramorum* [45]*.* The motif streme-5 (ATACCGGTAT; 10 nt) and motif-45 (TAATATWAC; 9 nt; TATA-like) were identified upstream of a set of 206 and 75 genes, respectively, which are enriched with RxLRs, proteases and transcription factors. These motifs correspond to motif-9 (GTACCGGTA; 9 nt) and motif-7 (TACTATTAGTA; 11 nt) respectively, found in the study of Seidl et al. [24].

### Comparison with other eukaryotes

The 4–16 nt long motifs identified in this study for 53 clusters occur in 2.15 to 100% of the genes of the corresponding clusters. In total, 61 cluster-unique motifs were identified using MEME and STREME (Additional files 4 and 5). In relation to expression-based cluster motif analysis performed in *Plasmodium falciparum* [26], the motif streme-13 (CAGCAACATC; 10 nt) found in this study was observed to be similar with CAAC-motif sets identified in the upstream regions of the DNA replication machinery set in *Pd. falciparum*. Additionally, the C-rich motif streme-11 (ARCCCCGYTRCA; 12 nt) identified in 60 sequences is similar to motifs (CCCCAT, TTCCC) upstream region of set of 15 mitochondrial genes in that species. The streme-1 motif (DRTGTGGWCCACAYH; 15 nt), which occurred in upstream region of 379 genes, is similar to the TGTG and CACA motif-sets, identified in the *Pd. falciparum* upstream regions of genes coding for genes involved in the organelle translation machinery and the proteasome**.** In this study, streme-6 (ATGGCTACA; 9 nt) and streme-12 (AACTTCAAC; 9 nt), were found to bear similarities with the most overrepresented motif E2F (TGGCGCCA; 8 nt) associated with DNA replication and glycolysis and a motif (KAACTA; 6 nt) of unknown function in the protist parasite *Cryptosporidium parvum* [58]. In addition, the second-most overrepresented motif-13 (AAACGAGAAT; 10 nt) of cluster 14, located at 27 out of 218 sequences, had previously been reported as GAGA-motif. As reported by Vela-Corcía et al. [59], the motifs motif-8 (AAATCAAAAC; 10 nt), motif-10 (ATTGGCTRAAA; 11 nt), motif-26 (CTTTTGAC; 8 nt), motif-31 (ATGATTGGCT; 10 nt), as well as motif-32 (ATGATTGACGA; 11 nt) bear similarities with the TTGNC consensus sequence for σ^70^-family factors of validated promoters of the *Pseudomonas aeruginosa* PA14 genome.

### Pathogenicity-related genes

A total of 381 putative pathogenicity-related genes, including 175 coding for putative RXLR effectors and 26 for putative Crinklers (CRNs) containing a secretion signal were identified in the RNA-seq gene clustering analysis. In a study on *Plasmopara viticola*, PvRxLR67 and some other effectors were upregulated 1 dpi, but were also active in later infection stages in a high virulence strain [60]. In the current study, CEG37612 (cl 14; IF phase; log2FC 12.0; dEER-like motif position (DEI): 30), CEG45621 (cl 8; IF phase; log2FC 11.7; RxLR-like motif position (RNLV): 27; dEER like motif position (EER): 36), and CEG49100 (cl 14; IF phase; log2FC 9.3; RxLR-like motif position (KLLR): 40; dEER like motif position (EER): 52) were significantly higher expressed as compared comparison to other putative RxLR effector encoding genes, suggesting an essential role in pathogenicity that could be explored in future studies.

Among 27 secreted putative CRN effectors, two highly expressed Crinkler genes were CEG37824 (cl 24; SP phase; log2FC 3.5; HVLVVVP-position: HVLVVVP-514) and CEG47474 (cl 7; IF phase; log2FC 4.2; HVLVVVP-position: HVLVELP-189). During the infection establishment, gene CEG37265 (cl 60; IF phase; log2FC -5.3; LFLAK-Position: LYLAK-47) has been down regulated to the largest extent. Stam et al. [61] classified CRNs into two classes on the basis of expression patterns. Genes with no detectable expression in the early stages and increase after the infection establishment (class 2 according to Stam et al. [61]) are CEG40812 (cl 11; LFLAK Positions: LFLAK-14), CEG42045 (cl 2; LFLAK-Position: EFLAN-92; HVLVVVP-position: HVLVVVP-302), and CEG49843 (cl 25; HVLVVVP-position: HVLVVIP-104). This suggests, that these genes are probably important in maintaining the infection process after establishment.

Pathogens secrete proteases and protease inhibitors to manipulate host defense for a successful colonization [62]. In this study 27% of the proteases were predicted to be secreted and, thus, have a potential role in pathogenicity. The genes found to be most up-regulated are CEG48424 (metalloprotease family) in cl 8, IF phase, log2FC 9.4, while CEG35751 (Cytosolic Ca2-dependent cysteine protease (calpain), large subunit (EF-Hand protein superfamily) in cl 11, IF phase, log2FC − 6.1 was most down regulated. The motifs streme-4 (TCATTYTYMAATTTK; 15 nt; putative INR + FPR) and streme-10 (GCCAAYCA; 8 nt; putative CCAAT box) regulating the genes transcription in cl 8 and cl 11 were found. This situation is similar to findings on proteolytic enzymes in the oomycete fish pathogen *Sa. parasitica* where seven and predicted secreted proteases were found to be expressed during infection [63, 64].

Furthermore, the secreted protease inhibitor coding genes highly up-regulated are CEG40179 (cl 29, SP phase, log2FC 7.87) and CEG36013 (cl 14, IF phase, log2FC 11.6). The expression patterns of the genes are in opposite direction to each other, even though they share a common motif streme-4 (TCATTYTYMAATTTK; 15 nt; putative INR + FPR) suggesting that other TF binding sites are more important for the regulation of the genes.

Only two genes encoding an ATP-binding cassette (ABC) transporter were identified in the clusters, CEG48435 (ABC transporter ATP-binding protein) and CEG37981 (ABC transporter e family member 2), in cl 12 and cl 59, respectively. CEG48435 and CEG37981 genes were increasingly up-regulated during infection, to the maximum extent in the late infection stages and expression down regulated on the induction of sporulation. The upstream motifs streme-1 (DRTGTGGWCCACAYH; 15 nt; TGTG+CACA like), streme-3 (GATTGGHTRAAAWDW; 15 nt), streme-4 (TCATTYTYMAATTTK; 15 nt; putative INR + FPR), streme-10 GCCAAYCA; 8 nt; putative CCAAT box) have been found common in cluster 12 and cl 59. There are recent findings that suggest these transporters might be involved in the detoxification of phytoalexins [65, 66].

### Ion channels

In the beginning of the infection (IF) phase the voltage-gated ion channels identified are generally downregulated but upregulated during CO and SP phase. An example of this is CEG38124 (Chloride channel family) in cl 11; CO phase; log2FC 5.94, which has been suggested to have importance in cell volume regulation [67] and, thus, might be an important component of plasma extension leading to sporulation.

### Plant cell wall degrading enzymes (PCWDEs)

Oomycetes express enzymes capable to dissolve or soften plant primary cell walls for initial penetration and the formation of haustoria. CAZyme coding genes were reported in, e.g. *Phytophthora* and *Plasmopara viticola* [68, 69]. A total of 27 genes coding for secreted carbohydrate-active enzymes (CAZyme) were found to be significantly upregulated at some stages of infection. These included carbohydrate esterases, glycoside hydrolases, glycosyl transferases and polysaccharide lyases (Additional file 9). In this study, the highly up-regulated and secreted-protein coding gene CEG46932 (a glycoside hydrolase family 6 protein) in cl 6 (IF phase; log2FC 9.5) had its maximum expression in the beginning of the infection phase (Additional files 5 and 9). This expression profile indicated a potential role in host penetration by degradation or modification of polymerous carbohydrates within the host cell wall.

### Transcription factors

The strong upregulation of some genes coding for transcription factors, e.g. CEG35443 (myb-like DNA-binding) in cl 1, IF phase, log2FC7.8 and CEG42655 (Transcription factor IIB) in cl 2, CO phase, log2FC 6.7 suggests their possible involvement as regulators of the infection and colonization processes, respectively. Further functional studies and validations are needed to elucidate the significance of these transcription factors in host-pathogen interaction.

## Conclusions and outlook

High-throughput RNA sequencing is an important tool for linking gene expression and function with potential regulatory motifs. In this study 41 novel and 169 known motifs were found in clustered gene groups with 16 to 1158 members. Some motifs were observed from multiple clusters suggesting a more general role in the cellular processes of *Pl.halstedii*. To the best of our knowledge, the identified putative novel motifs and known motifs except for streme-4 (TCATTYTYMAATTTK; 15 nt; putative INR + FPR), streme-10 (GCCAAYCA; 8 nt; putative CCAAT box) and streme-12 (AACTTCAAC; 9 nt; CAAC-like), have not been reported previously from oomycetes. Interestingly, some motifs such as streme-1 (DRTGTGGWCCACAYH; 15 nt; TGTG+CACA like) represented condensations of previously identified motifs. This might imply targeting by two independent transcription factors, probably as a possibility for regulation by competitive binding. However, it is clear that the current study only provided a first glimpse into genes with putative virulence functions and the diversity of potential transcription factor binding sites identified by motif searchers. This will need to be followed up by laboratory experiments to verify the importance of the highly differentially expressed genes in pathogenicity and to identify the potential functional redundancy of highly similar motifs and the potential transcription factors binding to the various motifs found. In the context of host-pathogen interactions, a systems biology view for host pathogen interaction could be a powerful means for achieving a system-wide understanding of cellular processes involved in pathogenicity. However, it should be noted that in the current study, only the 100 nt upstream of genes were investigated for core promoter motifs to enable a broad screening for potential motifs. Many core promoters will not have been fully covered by this approach, and an extension to 500 bp or more upstream of genes would likely lead to the discovery of additional regulatory motifs.

## Supplementary Information

**Additional file 1.**

**Additional file 2.**

**Additional file 3.**

**Additional file 4.**

**Additional file 5.**

**Additional file 6.**

**Additional file 7.**

**Additional file 8.**

**Additional file 9.**

**Additional file 10.**

## Data Availability

All data used in this study are available from GenBank under the accession number PRJEB49134 (https://www.ncbi.nlm.nih.gov/bioproject/49134).

## Abbreviations

avg.: Average
FC: fold change
bp: base pair
nt: nucleotides
DEGs: differentially expressed genes
TFBS: transcription factor binding site
cl: Cluster
